# Empowering Women and Improving Female Reproductive Health through Control of Neglected Tropical Diseases

**DOI:** 10.1371/journal.pntd.0000559

**Published:** 2009-11-24

**Authors:** Peter J. Hotez

**Affiliations:** 1 Department of Microbiology, Immunology, and Tropical Medicine, The George Washington University, Washington, D. C., United States of America; 2 Sabin Vaccine Institute, Washington, D. C., United States of America

Secretary Hillary Clinton has made the rights of women, especially those living in low-income countries, a central theme of her tenure at the United States Department of State. During her recent 11-day trip to sub-Saharan Africa, issues of gender equality and empowering women arguably had their highest profile ever [1], and there is a clear commitment by both the Secretary and the Obama administration to work aggressively toward achieving the two major Millennium Development Goals (MDGs) that specifically advocate for women, namely MDG 3 “Promote gender equality and empower women” and MDG 5 “Improve maternal health” (see http://www.un.org/millenniumgoals). Because the neglected tropical diseases (NTDs) are the most common infections among the world's poorest people [2], including girls and women, there is now a strong case to be made for controlling the NTDs as a means of directly addressing MDG 3 and MDG 5. Studies conducted over the last two decades provide an evidence base that the NTDs are important factors that (i) impair reproductive health in developing countries; (ii) increase the transmission of sexually transmitted infections (STIs); and (iii) promote stigma and gender inequality (Table 1). For these reasons, interventions focused on NTD control and elimination could offer an opportunity for improving the health and rights of girls and women in the poorest countries of Africa, Asia, and Latin America and the Caribbean.

**Table 1:** Health Threats to Women Resulting from Neglected Tropical Diseases.

Health Condition	Neglected Tropical Disease	References
**Reproductive Health**		
Infertility	Urogenital schistosomiasis, hookworm	[3], [16]–[18]
Severe anemia of pregnancy/lactation and high maternal morbidity and mortality	Hookworm (major), schistosomiasis (minor)	[3]–[13]
Anemia associated with menstruation and amenorrhea	Hookworm	[3],[6]
Congenital infection; lactogenic infection	Chagas disease, leishmaniasis, strongyloidiasis, hookworm	[6], [22]–[28]
Low birthweight and/or premature birth from placental inflammation and maternal anemia	Hookworm and other soil-transmitted helminth infections, schistosomiasis	[10]–[13]
Exacerbation of disease during pregnancy	Leprosy, schistosomiasis	[29],[30]
**Sexually Transmitted Infections**		
HIV/AIDS	Urogenital schistosomiasis	[33]–[35]
Trichomoniasis	Trichomoniasis	[31],[32]
**Social Exclusion and Stigma**		
Limb, breast, skin, and genital deformities	Lymphatic filariasis, Buruli ulcer, *Onchocerca* skin disease, leprosy, leishmaniasis	[36]–[45]
Facial disfigurement	Leishmaniasis, leprosy	[36], [46]–[48]

## The Impact of NTDs on Female Reproductive Health

Pregnancy and lactation place huge iron demands on the mother and her child. A 1994 report from the World Health Organization (WHO) concluded that a woman living in a developing country is practically always on the verge of iron deficiency anemia either because of pregnancy, which requires the transfer of 300 mg of iron to the fetus during the third trimester and an additional 500 mg of iron to accommodate an increase in red blood cell mass, or lactation, in which each episode transfers 0.75 mg of iron from mother to child [3]. Moreover, even before she becomes pregnant, a woman of childbearing age suffers substantial iron losses from menstruation [3]. Anemia, defined as a reduction in hemoglobin to <11 g/dl in the first and third trimester and <10.5 g/dl in the second trimester, creates a dangerous state of health for both mother and child [4]. It is estimated that 20% of maternal deaths in Africa are attributed to anemia, while simultaneously anemia represents a key risk factor for poor pregnancy outcome and low birth weight [4],[5]. It now appears that human hookworm infection, one of the most common NTDs affecting 576–740 million people in developing countries, considerably adds to the iron loss and anemia that occurs during pregnancy [6]. An estimated 44 million pregnant women are infected with hookworm at any one time [3], including up to one-third of all pregnant women in sub-Saharan Africa [7]. In Africa and Latin America, hookworm is a major contributor to anemia in pregnancy [7],[8], while in Nepal and presumably elsewhere in Asia hookworm is responsible for 54% of cases of moderate to severe anemia during pregnancy [9]. Not surprisingly, deworming during pregnancy has major beneficial effects in terms of reduced maternal morbidity and mortality, as well as improved perinatal outcome [10],[11], and most likely leads to a reduction in maternal anemia. Such studies have led to calls for including deworming in antenatal packages in hookworm-endemic areas in developing countries [5],[6],[12].

There is also some evidence that schistosomiasis in pregnancy contributes to increased maternal morbidity and low birth weight [13]. Like hookworm infection, schistosomiasis is an important cause of anemia in Africa [5],[14], but in addition, schistosome eggs can be deposited in the placenta where they cause inflammation, and this feature may also contribute to adverse maternal-fetal outcomes [13]. Therefore, there is a need for expanded studies of praziquantel administration during pregnancy to complement the studies purporting a beneficial effect of anthelmintic drugs for hookworm and other soil-transmitted helminth infections.

In addition to adverse pregnancy outcomes, both hookworm infection and schistosomiasis contribute to infertility. Since the 1920s, it has been noted that chronic hookworm among women of reproductive age causes amenorrhea and sterility, and that both regular menses and fertility could often be restored through deworming [3]. In sub-Saharan Africa there are an estimated 112 million people infected with urinary tract schistosomiasis caused by *Schistosoma haematobium* [15]. Up to 75% of women with *S. haematobium* infection are also at risk for infertility because of genitourinary schistosomiasis caused by the deposition of schistosome eggs and the resulting granulomatous inflammation in the uterus, fallopian tubes, and ovaries [16]–[18]. There is interest in potentially preventing the onset of these inflammatory processes through early intervention with praziquantel [19]–[21].

It has also been noted that congenital infections with some NTD pathogens can occur commonly. Congenital toxoplasmosis and malaria are the best-known examples [22],[23], but there is also now evidence that congenital Chagas disease occurs with high frequency among seropositive pregnant mothers, particularly those with parasitemia [24],[25]. Congenital leishmaniasis has also been described [26],[27], as has lactogenic infection of hookworm and strongyloidiasis [6],[28]. Finally, pregnancy can result in host immunomodulatory effects that could affect the severity of both schistosomiasis, leprosy, and presumably other NTDs [29],[30].

## NTDs and STIs

Several NTDs are also STIs or, in some cases, NTDs promote susceptibility to other STIs. For example, trichomoniasis is both an NTD and an STI with the parasitic protozoan *Trichomonas vaginalis*, which is now recognized as one of the most common STIs in Africa and elsewhere [31]. In the United States of America, trichomoniasis is also an important STI among poor and under-represented minority populations [32]. Female genital schistosomiasis (especially of the lower genital tract) has been identified as an important co-factor in HIV transmission in rural areas of Africa where *S. haematobium* and HIV/AIDS are co-endemic [16], [33]–[35]. It has been suggested that the schistosome egg granulomas function as erosive or ulcerative lesions in the cervix and vagina, possibly similar to those caused by other STIs such as herpes simplex virus-2 infection or syphilis. Such lesions presumably facilitate HIV viral entry, or possibly HIV entry, and replication is enhanced by the propensity of the schistosome egg granulomas to cause bleeding or serve as a repository of CD4+ cells [16]. In any case, female genital schistosomiasis has been shown in a rural area of northern Zimbabwe to be associated with a 3-fold risk of horizontal HIV/AIDS transmission [34], furthering the urgency to investigate praziquantel treatment of this condition as a part of a larger allied effort toward HIV/AIDS prevention.

## NTDs, Stigma, and Gender Inequality

In her 2007 address to the WHO Global Partners Meeting on NTDs, Margaret Chan, the Director-General of WHO, stated that “stigma and social isolation, especially for women, compound the misery and further embed people in poverty” [36]. Previous articles in *PLoS Neglected Tropical Diseases* have explored the important social and economic consequences of stigma associated with the disfigurement of many NTDs, including lymphatic filariasis (LF), onchocerciasis, leishmaniasis, and the mycobacterial infections (such as Buruli ulcer and leprosy [37],[38]), and indeed, emerging evidence (summarized in a 2005 report commissioned by WHO-Special Programme for Research and Training in Tropical Diseases [TDR]) suggests that women are often particularly isolated and marginalized by stigma-associated NTDs [39]. In LF, lymphedema occurs more frequently in women than in men [39],[40], often with involvement of the breasts and vulva [39],[41], but these clinical features frequently go unnoticed because in some developing countries the examination of women is restricted to the arms and legs [39],[42]. A recent qualitative study of LF from Sri Lanka has articulated the breadth and depth of social stigma linked to LF among women and includes evidence for lost jobs and wages and abandonment by family [38]. Similarly, African women are disproportionately ostracized for *Onchocerca* skin disease [43]–[45], and in South Asia women are sometimes prevented from seeking medical attention for kala-azar (accounting for a higher disease burden from this condition among women) [46]. In Afghanistan, cutaneous leishmaniasis prevents mothers from holding their children, while in Colombia this disease is a contributing factor for spousal abandonment [46]. The social impact of leprosy is also greater among women [39],[47],[48], an observation that stimulated the WHO Director-General to remark in 2007, “imagine the impact when a young woman with leprosy is told she can be fully cured, can marry, have children, and will not infect others. Just imagine the impact” [36].

## Future Directions

Because of the dramatic impact of NTDs on the health of women, especially girls and women in their child-bearing years, it is critically important that these populations are included in current and proposed large-scale interventions for NTDs. Efforts to expand global deworming with benzimidazole anthelmintics against soil-transmitted helminth infections and praziquantel against schistosomiasis should include pregnant women as recommended in recently issued WHO guidelines for helminth control [49]. At the same time, there should be increased efforts to conduct safety testing of ivermectin and diethylcarbamazine in pregnancy or during lactation, such as the one study recently reported from Uganda [50], in order to ensure that all women in their reproductive years may one day become eligible for mass drug administration against onchocerciasis and LF, as well as for integrated control against all of the most common NTDs [2]. Similarly, there is a need for additional operational research on the beneficial effects of NTD control on pregnancy outcome, and studies to examine the impact of praziquantel and possibly other anthelmintics on reducing HIV/AIDS transmission among women in their reproductive years. Additional social science research on gender inequalities for NTDs is also urgently needed. Finally, it has been pointed out that in developing countries women are “key agents of change” whose role could be expanded to further promote social mobilization, including ensuring compliance in community-based drug distribution and treatment programs for NTDs as well as in vector control [51]. Increasingly, the NTD community needs to enlist the support of women throughout the developing world as a critical part of ensuring access to essential medicines against the NTDs. Both the WHO's Department of Control of Neglected Tropical Diseases and a new Global Network for Neglected Tropical Diseases have placed the empowerment of women as a top priority in its pursuit of widespread coverage for the most common NTDs, including soil-transmitted helminth infections, schistosomiasis, LF, and onchocerciasis [2], while new initiatives devoted to research and development by WHO-TDR are currently championing gender issues [39]. Simultaneously, both WHO-TDR and nonprofit product development partnerships, including the Human Hookworm Vaccine Initiative through the Sabin Vaccine Institute, will champion the inclusion of women in the clinical development of new drugs, diagnostics, and vaccines. Such activities create a robust opportunity to prioritize the control of NTDs and NTD research and development and designate these activities as key enabling mechanisms for advancing women's reproductive and maternal health.
